# Assessment of Clinical Reasoning During a High Stakes Medical Student OSCE

**DOI:** 10.5334/pme.1513

**Published:** 2024-12-12

**Authors:** Jeffrey Siegelman, Lisa Bernstein, Jennifer Goedken, Linda Lewin, Jason Schneider, Martha Ward, Hugh Stoddard

**Affiliations:** 1 Department of Emergency Medicine, Emory University School of Medicine, Atlanta, Georgia, US; 2 Department of Medicine, Emory University School of Medicine, Atlanta, Georgia, US; 3 Department of Gynecology and Obstetrics, Emory University School of Medicine, Atlanta, Georgia, US; 4 Department of Pediatrics, Emory University School of Medicine, Atlanta, Georgia, US; 5 Departments of Medicine and Psychiatry and Behavioral Sciences, Emory University School of Medicine, Atlanta, Georgia, US; 6 Department of Education, Innovation, and Technology, Baylor School of Medicine and Assistant Dean for Medical Education Research, US

## Abstract

**Background & Need for Innovation:**

Objective Structured Clinical Examinations (OSCEs) are commonly employed to assess clinical skills. While several existing tools address components of clinical reasoning, including the Assessment of Reasoning Tool, none are calibrated for competency-based assessment of medical students (UME) in an OSCE setting.

**Goal of Innovation:**

We sought to create a clinical reasoning assessment for use in a high-stakes, summative medical student OSCE.

**Steps taken for Development and Implementation of innovation:**

A minimum-competency OSCE was administered to medical students following their required clinical clerkships. We developed a tool to assess clinical reasoning of medical students at the conclusion of their required clinical clerkships and deployed it during a minimum-competency OSCE exam given at that time. The highest level of the modified tool represented minimum acceptable performance for examinees.

**Evaluation of Innovation:**

The scores and analyses provided evidence to support the use of this tool. Examinees’ performance on clinical reasoning tasks was comparable with their overall performance on the OSCE. The sub-scores for clinical reasoning accurately distinguished successful examinees from those who did not meet the minimum performance level, providing support for the use of the tool in this high stakes setting.

**Critical Reflection:**

This tool was found to be useful and defensible to assess medical students’ clinical reasoning. Expanded evidence for generalizability of the tool and its utility in other settings will need to be garnered through multi-center implementation and study.

## Background & Need for Innovation

Clinical reasoning, the iterative process of gathering information, generating a problem representation, recognizing patterns, comparing and contrasting possibilities to identify a leading diagnostic hypothesis, and prioritizing patient evaluation and management, is an integral skill of the practicing clinician [1,2]. In preparing medical students for transition to residency training, robust assessment of both diagnostic and management reasoning skills is essential [3,4]. In the United States, the national, standardized clinical skills examination was discontinued in 2020 [5], placing the onus for clinical reasoning assessment on medical schools.

Several tools are available to assess trainee clinical reasoning through written or oral examinations, bedside assessments, or simulation, with each tool having some capacity to capture individual components of the skill [2]. Objective Structured Clinical Examinations (OSCEs) offer greater clinical authenticity than written or oral examinations while also allowing for more controlled and standardized scenarios than are possible at the bedside using workplace-based assessments [6].

It is challenging, however, to choose an assessment method that captures the learners’ reasoning and is efficiently deployed in an OSCE setting. While oral patient presentations might elucidate learner thinking, scoring them is complicated and resource intensive. A student-generated note, based on a standardized patient (SP) encounter, balances authenticity with feasibility to provide insight into clinical reasoning without unduly complicating the OSCE administration.

Thammasitboon, with colleagues from the Society to Improve Diagnosis in Medicine, developed the Assessment of Reasoning Tool (ART) for use with trainees [1]. The ART is a structured instrument that reflects the key steps in the clinical reasoning process for oral presentations. The same team later published validity evidence for the tool using animated simulations of performance at various competency levels to which faculty raters applied the ART [7].

Although the ART is robust and comprehensive, it was neither tailored to assess learners in undergraduate medical education nor was it scored in a manner to produce a dichotomous determination of satisfactory or unsatisfactory performance. We valued the ability to use the ART as a theoretical framework from which we could innovate a tool more suitable for use in an OSCE examination to make a competency-based determination.

## Goal of Innovation

We sought to develop and examine a new tool for use with senior medical students in a high-stakes OSCE to gather evidence of its effectiveness for making justifiable, summative determinations about clinical reasoning skills.

## Development and Implementation of Innovation

A team of experienced clinical educators developed the assessment tool with guidance from an assessment specialist. We adopted the theoretical characterization of clinical reasoning based on the theories of semantic networks described by Bordage [8] and behavioral descriptors from the ART to inform the new tool. We, however, modified the performance levels of the tool to an ordinal scale (0, 1, or 2) where a score of 2 denoted an examinee who clearly met the minimum acceptable level of performance (i.e. Pass), while scores of 1 and 0 denoted borderline and substandard performance (i.e. Fail), respectively.

We constructed descriptive anchors for each performance level using terminology from the original ART (Table 1) and aligned them with our program’s learner outcomes [9]. We applied the preliminary tool to existing OSCE videos, discussed and iteratively revised it through discussion to ensure consistent interpretation among raters. Specifically, we removed items relating to metacognition because we could not represent this construct in the patient note. We also removed other clinical skills ART items, such as history-taking and physical exams skills since other instruments measured these during the same OSCE.

**Table 1:** Domains Measured on Original ART and New Assessment Tool.


ASSESSMENT OF REASONING TOOL	NEW ASSESSMENT TOOL

Hypothesis-directed data collection	Not assessed (measured elsewhere on OSCE)

Problem representation	Problem representation

Prioritized differential diagnosis	Prioritized differential diagnosis

Direct evaluation/treatment towards high priority diagnoses	Initial management plan (as expected for graduating medical student)

Metacognition	Not assessed


OSCE: Observed Structured Clinical Examination.

Our medical students must successfully complete a four-station OSCE after finishing their core clinical clerkships. During each station the examinee must perform a focused history and physical exam, communicate effectively, manage a challenging conversation with the SP, and then compose a patient note demonstrating their clinical reasoning process (Appendix 1). The same team members who developed the tool scored the patient notes after receiving specific training. The team discussed the tool’s language and grading philosophy during bi-weekly meetings to ensure appropriate calibration across all cases and raters.

Raters scored each examinee’s patient notes on three clinical reasoning components: constructing a prioritized differential diagnosis, providing a problem representation, and proposing next steps for patient evaluation and/or care (Appendix 2). The scores for each component of the 4 SP encounters were summed to create a total clinical reasoning score. This total score was compared to a cut score that had been previously defined using the Hofstee method [10] with pilot data to make a pass or fail determination for the task of clinical reasoning. Similarly, scores for four discrete OSCE tasks (history-taking, physical examination, communication, and difficult conversations) were calculated from other tools to create a total score for each task, although these scores did not directly affect clinical reasoning scores. Although clinical reasoning is content and context specific, for the purposes of student assessment in a minimum-competency OSCE, the composite score was expeditious for representing an overall understanding of students’ performance on the exam.

The original ART [7] offered foundational validity evidence for our new tool. Content validity was established based on development of the tool from a well-studied instrument and expert review of the new items by the author group. Response process was established based on collaborative development of clear anchors for scoring and discussion among the raters during tool development. We present subsequent validity evidence below.

This study was approved by the Emory Institutional Review Board, reference number 00004028.

## Evaluation of Innovation

136 fourth-year medical students completed this OSCE in 2021, and we used this un-sampled cohort for analysis.

We conducted two distinct analyses to support the use of this new clinical reasoning assessment tool within the OSCE context. The first analysis evaluated how well the clinical reasoning scores aligned with other foundational clinical skills assessed during the same OSCE. The second analysis examined the convergence of the new tool by correlating the individual sub-scores from three clinical reasoning tasks and the overall clinical reasoning score.

We employed Analysis of Variance (ANOVA) techniques for both analyses rather than correlation or regression methods. This choice was guided by our primary objective: validating pass/fail decisions for minimum competency assessment. The ANOVA allowed us to focus specifically on examinees who achieved passing status as a cohesive group and avoid the confounding influence of within-group performance variations that were not relevant to pass/fail decisions. This methodological decision excluded extraneous variations in performance among passing candidates that would have been incorporated into correlation or regression analyses. Please consider this:

Our first analysis (Table 2) demonstrated strong alignment between examinees’ performance on individual clinical reasoning tasks and their overall examination performance. Of particular significance for a minimum competency pass/fail examination was the performance pattern of the Borderline group, which proved crucial in validating both the new tool and established cut scores. Performance across individual tasks closely aligned with the overall performance in the OSCE. Notably, for three skills – history-taking, physical examination, and communication – ANOVA comparisons revealed that the Borderline group’s performance more closely resembled the Fail group than the Pass group. These three tasks, representing foundational skills acquired early in medical training, served as particularly effective discriminators of minimum competency.

**Table 2:** Mean score for each of the overall performance groups on each of the OSCE tasks.


		MEAN TASK SCORE FOR EACH PERFORMANCE GROUP				

		HISTORY TAKING	PHYSICAL EXAMINATION	CLINICAL REASONING	COMMUNICATION	DIFFICULT CONVERSATION

Examinee Group Based on Overall Performance in EOA OSCE	Pass (n = 106)	89.6%	83.2%	86.2%	94.1%	87.9%

	Borderline (n = 15)	85.3%	79.1%	83.6%	88.6%	72.3%

	Fail (n = 15)	83.2%	78.1%	68.6%	86.3%	65.0%

	ALL Examinees (n = 136)	88.40%	82.16%	83.98%	92.65%	83.66%

	*Eta*^2^ *effect size*	*0.227*	*0.101*	*0.191*	*0.239*	*0.562*


Scores are highlighted for instances when a group differed from other groups on a specific task (ANOVA [F(2,133)]; p < .05). Mean scores for History Taking and Physical Examination were calculated as the percentage of skills completed based on a validated checklist for each case. Mean scores for Communication and Difficult Conversations were calculated as a percentage of total available points based on a rating scale that was designed and validated for each case. Abbreviations: ART – Assessment of Reasoning Tool, EOA OSCE – End of Applications Objective Structured Clinical Encounter, ANOVA – analysis of variance.

The second analysis, (Table 3) illustrated the relationship of the clinical reasoning sub-scores and the total clinical reasoning scores. All sub-scores followed the expected hierarchical pattern, with higher performing groups consistently achieving better sub-scores across all tasks of clinical reasoning. The “problem representation” sub-score emerged as a particularly robust discriminator showing statistically significant differences (p < .05) between all three performance groups. This finding suggests that problem representation skills may be a key indicator of overall clinical reasoning competency.

**Table 3:** Mean sub-score for each of the overall performance groups on the 3 clinical reasoning components.


		MEAN SUB-SCORE FOR EACH PERFORMANCE GROUP		

		DIFFERENTIAL DIAGNOSIS	PROBLEM REPRESENTATION	NEXT STEPS

Examinee Group Based on Overall Performance on the Clinical Reasoning Task	Pass (n = 107)	92.6%	87.9%	87.5%

	Borderline (n = 22)	79.5%	43.8%	78.4%

	Fail (n = 7)	75.0%	12.5%	76.8%

	ALL (n = 136)	89.61%	76.84%	85.48%

	*Eta*^2^ *effect size*	*0.257*	*0.641*	*0.097*


Scores are highlighted for instances when a group differed from other groups on a specific component (ANOVA [F(2,133)]; p < .05). Abbreviations: ART – Assessment of Reasoning Tool, EOA OSCE – End of Applications Objective Structured Clinical Encounter, ANOVA – analysis of variance.

The combined analyses provide empirical evidence to support the use of this new assessment tool in evaluating minimum competency in clinical reasoning among senior medical students.

## Critical Reflection on the Process

We created and implemented an assessment tool, grounded in the ART framework, to determine minimum competency in clinical reasoning among 136 senior medical students during a high-stakes OSCE. Building on the established ART framework, which had previously demonstrated internal consistency and positive correlation with global learner assessments of oral presentations [6,7], we adapted the tool for our specific assessment needs.

Initial evaluations yielded promising results, with our new tool showing positive correlations across several foundational clinical skills tasks, including history-taking, communication, and management of difficult conversations. While the internal consistency for clinical reasoning assessment was overall borderline, the “problem representation” sub-score demonstrated adequate internal consistency, suggesting this as a particularly robust element of the assessment.

The implementation process has enhanced our ability to comprehensively assess clinical reasoning at our institution, though opportunities for improvement remain. To strengthen the tool’s effectiveness, we have initiated collaborative discussions with clerkship and course directors to ensure alignment between pre-clerkship instruction and clinical experiences, particularly regarding problem representation concepts. Our experience indicates that while current rater training protocols are adequate, additional training will be necessary to improve consistency in evaluating other components of clinical reasoning. Point biserial correlations for the three components indicate that the component sub-scores are effective to distinguish between high-performing and low-performing students.

Given the universal importance of clinical reasoning across health professions, this tool holds potential value for standardized high-stakes assessment in various health profession education contexts. However, successful implementation requires careful alignment between curricular content and assessment methods, particularly in teaching prioritized differential diagnosis, problem representation statements, and basic management plans. While these initial findings are encouraging, we acknowledge the limitations of our single-institution study. Further validation studies across multiple institutions and diverse OSCE contexts will be necessary to fully establish the tool’s reliability and generalizability. Additional research should focus on enhancing inter-rater reliability and validating the tool’s effectiveness across different healthcare education settings.

## Conclusion

Our new tool shows promise for assessing clinical reasoning competency for senior students in high-stakes OSCEs as medical schools strive to verify graduates’ readiness for residency training. While initial results demonstrate the tool’s effectiveness for minimum competency decisions, multi-center implementation studies are needed to establish broader generalizability and justify its use in summative assessment. Future development should focus on enhancing inter-rater reliability and validating the tool across diverse educational settings, supporting its transition from formative to high-stakes evaluation contexts.

## Additional Files

The additional files for this article can be found as follows:

10.5334/pme.1513.s1 Appendix 1. Supplemental Digital Appendix 1.

10.5334/pme.1513.s2 Appendix 2. Supplemental Digital Appendix 2.
