# Supplementary material for: Assessment of Clinical Reasoning During a High Stakes Medical Student OSCE
Source: Perspect Med Educ. 2024 Dec 12;13(1):629–34. doi: 10.5334/pme.1513 (PMC11639687; doi:10.5334/pme.1513)
Supplement: Appendix 1. — Supplemental Digital Appendix 1. [file pme-13-1-1513-s1.pdf]

Supplemental Digital Appendix 1

Learner note template as viewed in Learning Space

Post-Encounter Learner

Note Instructions (Note Inst.)

NOTE INSTRUCTIONS:

- **YOU HAVE 10 MINUTES TO COMPLETE ALL PARTS OF THE FOLLOWING WRITE-UP**
- **PLEASE READ THE DIRECTIONS ABOVE EACH SECTION CAREFULLY AS THE INSTRUCTIONS ARE DIFFERENT FOR EACH SECTION AND CASE.**
- **To add additional lines to each section, click the "add" button.**
- **Timer will start when you click inside a text field.**

GOOD LUCK!

Student Score SPA 4 (SPA 4)

21.
Provide the top two to four diagnoses on your differential, **with the most likely diagnosis listed first.**

Open-Ended Scoring

22.
Provide a concise problem representation for this case, including all pertinent positives and negatives from the H&P, to support your **most likely** diagnosis.

Open-Ended Scoring

23.
Based on your differential diagnosis, what diagnostic/therapeutic step(s) would you take today? (such as: laboratory, imaging, referrals, counseling, therapeutics)

Open-Ended Scoring
