# Supplementary material for: Assessment of Clinical Reasoning During a High Stakes Medical Student OSCE
Source: Perspect Med Educ. 2024 Dec 12;13(1):629–34. doi: 10.5334/pme.1513 (PMC11639687; doi:10.5334/pme.1513)
Supplement: Appendix 2. — Supplemental Digital Appendix 2. [file pme-13-1-1513-s2.pdf]

Supplemental Digital Appendix 2

EOA rubric seen by faculty in Learning Space for note grading

Problem Representation

1.<br>**Problem Representation**	2. Well organized problem representation which Includes key clinical findings (both positive and negative) and uses descriptive medical terminology to support most likely diagnosis.<br>[2]
	1.<br><br>[1]
	0. Does not write information in the form of a problem representation<br>[0]

Differential Diagnosis

1.<br>**Differential Diagnosis**	2. Provides a reasonable differential diagnosis with the most likely diagnosis listed first<br>[2]
	1.<br><br>[1]
	0. None or only 1 dx and that one is not the most likely<br>[0]

Next Steps

1.<br>**Next Steps based on differential**	2. Diagnostic and therapeutic plan is reasonable for the differential and focuses on immediate next steps<br>[2]
	1.<br><br>[1]
	0. Does not include next steps<br>[0]
